# Wisdom and Life Purpose as Predictors of Mental Well-Being Among Middle-Aged to Older Adults: Cross-Sectional Study

**DOI:** 10.2196/91716

**Published:** 2026-05-19

**Authors:** Ibrahim Arpaci, Ismail Kuşci, Kasim Karataş, Mustafa Baloglu

**Affiliations:** 1 Department of Engineering, Faculty of Engineering, Bursa Uludag Uni̇versi̇ty, Bursa, Turkey; 2 Department of Computer Science and Engineering, College of Informatics, Korea University, Seoul, Republic of Korea; 3 Department of Counseling, Faculty of Education, Tokat Gaziosmanpasa University, Tokat, Turkey; 4 Department of Educational Sciences, Faculty of Education, Karamanoğlu Mehmetbey University, Karaman, Turkey; 5 Special and Gifted Education, Faculty of Education, United Arab Emirates University, Sheik Khalifa Bin Zayed Street, Asharej, Al Ain, Abu Dhabi, United Arab Emirates, 971 501259095

**Keywords:** positive psychology, purpose in life, mental well-being, wisdom, aging

## Abstract

**Background:**

Positive aging, a concept found in positive psychology, serves as the theoretical foundation for this study. To age positively, one must manage hidden or unrecognized challenges, show flexibility in behavior and thought, adopt a positive outlook on problems involving regression, and make decisions that promote one’s well-being.

**Objective:**

This study examined the role of wisdom and life purpose in the mental well-being of middle-aged and older adults. More specifically, we tested 4 hypotheses: wisdom would exhibit a positive correlation with mental well-being, quality of life would exhibit a positive correlation with mental well-being, meaning and purpose would exhibit a positive correlation with mental well-being, and freedom would exhibit a positive correlation with mental well-being.

**Methods:**

The research used a multianalytical methodology combining covariance-based structural equation modeling and artificial neural network techniques to analyze data from 377 individuals aged 50 to 102 years.

**Results:**

Results from the covariance-based structural equation modeling indicate that meaning and purpose, wisdom, and quality of life were significantly associated with the mental well-being, accounting for 71% of the explained variance. Additionally, the artificial network analysis yielded exact forecasts of mental well-being. The artificial network model achieved an accuracy of 82.1% and 73% on the training and test sets, respectively, for predicting mental well-being. Sensitivity analysis revealed that meaning and purpose were the most critical factors in explaining participants’ mental well-being.

**Conclusions:**

These findings have prominent theoretical implications for social psychology researchers and practical consequences for authorities involved in the care of older adults, who can use the results to develop strategic plans and take necessary actions.

## Introduction

### Background

Worldwide aging is an inevitable demographic reality of the 21st century. The number of people aged 60 years and older was 962 million in 2017, and it is projected to double to 2.1 billion by 2050 [1]. Approximately 16% of the population in the United States, 20% in the European Union, and 27% in Japan are currently classified as older adults. The population of older adults is also increasing rapidly in many transitional countries, where the younger population is in the majority [2].

With advances in health care, life expectancy has increased significantly. Consequently, achieving a healthy and happy life in old age has become crucial. From a psychological perspective, aging involves knowledge gained through age, wisdom attained through experience, social relationships, future goals, coping with life events, life satisfaction, happiness, and the meaning of life [3]. Whereas traditional psychology aims to improve mental health, it often overlooks individuals’ strengths and well-being [4]. With the shift from modernism to postmodernism, significant changes have taken place, particularly in the humanities. Postmodernist approaches have explored the positive aspects of individuals and their well-being, leading to the emergence of positive psychology [5].

Well-being is discussed from 2 perspectives: pleasure (hedonic) and psychological functionality (eudaimonic) [6]. According to the hedonic point of view, the pursuit of pleasure, avoiding disturbances to one’s comfort, and avoiding anything that may cause negative feelings form the basis of subjective well-being [6]. Subjective well-being is also defined as experiencing more positive emotions and fewer negative emotions, thus having higher levels of life satisfaction [7]. The eudaimonic perspective, which represents the individuals’ attempts to realize their potential by acting in accordance with the essence, engaging in activities that express virtue, and achieving a good life because of internally motivated actions, is another source of well-being [8]. The eudaimonic approach is based on the principle that individuals accept themselves, communicate effectively with others, find meaning and purpose in life, and develop personally through environmental opportunities [6]. Because eudaimonic well-being is based on self-actualization, it is also associated with the meaning of life [6].

The World Health Organization defines mental well-being as “a state in which each individual reveals his or her potential, copes with the daily stresses of life, works productively and efficiently, and contributes to the society in which he or she lives.” Personality traits and environmental factors contribute to the development of subjective well-being, which underscores the importance of attending to developmental stages [9]. The concept of mental well-being used in this study includes both subjective well-being (ie, hedonic) and psychological well-being (ie, eudaimonic) dimensions.

Traditional psychology has primarily concentrated on enhancing mental health and addressing psychological problems. However, a growing body of research indicates that fostering positive traits and well-being may play a critical role in preventing mental health issues and promoting overall health and happiness. The hedonic perspective emphasizes comfort and pleasure, and the eudaimonic perspective focuses on self-actualization and a meaningful life. They are the two contemporary perspectives on well-being. Using a combined approach, we examine how wisdom and purpose in life can help explain mental health among middle-aged and older adults.

### Hypotheses and Theoretical Model

Positive aging is a concept of positive psychology and serves as the theoretical foundation for this study. To age positively, one must manage hidden or unrecognized challenges, show flexibility in behavior and thought, adopt a positive outlook on problems involving regression, and make decisions that promote one’s well-being [10]. Successful aging and positive aging are frequently used synonymously in the literature. Successful aging comprises several elements such as higher levels of mental and physical functioning, the avoidance of disease, active participation in life, financial security, life satisfaction, psychological well-being, and a positive outlook on life [10]. Chronological, social, biological, and psychological factors all contribute to successful aging. Similarly, positive aging refers to a wide range of factors that contribute to a happy and healthy life, including psychologically healthy behaviors, healthy lifestyle choices, interpersonal relationships, and the pursuit of meaning in one’s life [11].

**Figure 1.**
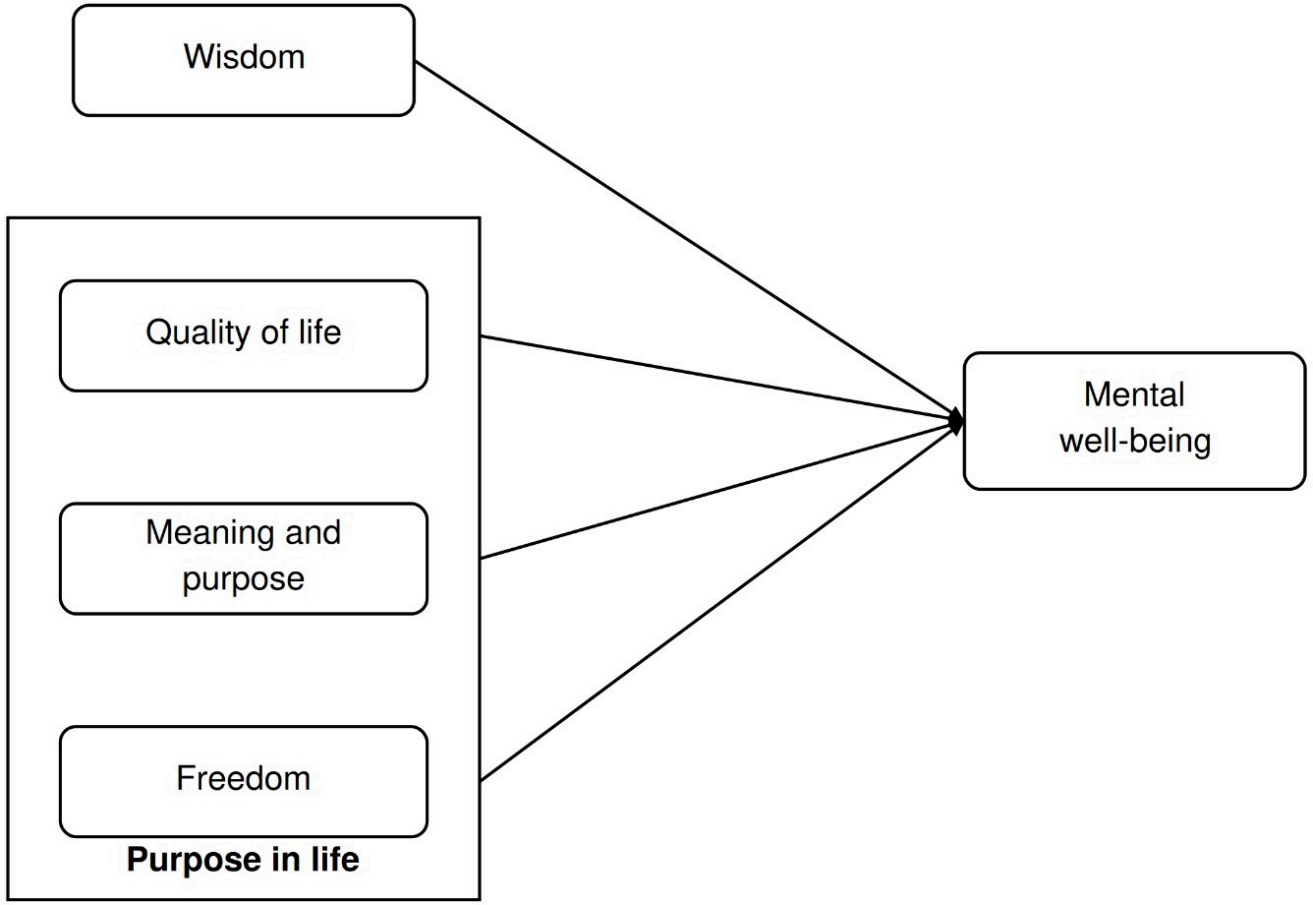
Theoretical model.

The increasing older adult population and the resulting social change have brought about a new social structure. As a result, research is needed to support this restructuring in ways that promote positive aging and help older adults live better, healthier lives. The development of interventions and programs for positive aging can be guided by considering the elements associated with positive aging. In the context of positive aging, we examined the connections among mental well-being, wisdom, and purpose in life (Figure 1).

### Wisdom and Well-Being

Wisdom is defined as the ability to make good decisions by using experience and knowledge and described as “the quality of having experience, knowledge, and sound judgment” or “the fact of relying on logical or intelligent reasoning” [3]. From a psychological perspective, wisdom is understanding and insight into the world and self, as well as the ability to make the right decisions when confronted with complex life problems [12]. Wisdom involves the application of skills and dispositions to promote the common well-being. Many cultures extol wisdom as an admirable resource that embodies the ideal harmony of action, knowledge, reason, and virtue [5]. It is perceived as the ultimate understanding of the human condition, along with a means of leading a good life and ending it well [13].

Due to its uniqueness, the wise can make better decisions by quickly analyzing situations and adapting to living conditions. Because wise individuals have a profound understanding of life, they can embrace its unpredictability and act calmly in the face of uncertainty, thereby reducing stress and contributing to well-being [14]. Wisdom has an impact on well-being as a psychosocial developmental resource [15]. Particularly in old age, wise individuals experience greater well-being because they know how to deal with loss [13]. Studies have shown a significant connection between wisdom and well-being. Wisdom increases well-being in older adults and enables them to actively take control of and learn from life rather than passively react to events. Moreover, wisdom is also positively associated with physical health [3]. Therefore, we proposed that wisdom would exhibit a positive correlation with mental well-being (hypothesis 1).

### The Quality of Life and Well-Being

The quality of life is defined in different ways, such as a response of the social environment; the intersection of individuals’ satisfaction with their social relations; or happiness, satisfaction, and harmony [16]. In the most general sense, the quality of life is the way individuals perceive life within the framework of their culture and values [17]. It is described according to 3 basic approaches. The first approach explains the quality of life in terms of normative considerations rather than idiosyncratic, the second links satisfaction with choices, and the third takes individual experiences into account [9].

The quality of life is influenced by psychological, physical, belief, socioeconomic, social, and environmental factors [17]. Well-being is one of those factors as well and is associated with a positive self, awareness of potentials and abilities, and acceptance of the self [8]. People with higher levels of well-being have better physical and mental health and have a higher standard of living. Well-being directly affects physical, mental, and emotional health, also affecting the quality of life [18]. Positive aging corresponds to cognitive, social, and emotional perspectives [19]. These are regarded as quality of life, functional care, and being healthy [19]. Well-being enhances quality of life, which is considered critical for the sustainability of positive aging [20]. Thus, we hypothesized that quality of life would exhibit a positive correlation with mental well-being (hypothesis 2).

### Meaning, Purpose, and Well-Being

Meaning is the feeling of belonging to something bigger than oneself [21]. People find meaning by having a sense of purpose and awareness [21,22]. When people find their own goals, they experience happiness and self-actualization. Well-being is associated with the meaning of life [6]. Meaning also increases positive emotions and provides a more satisfying life [23]. Personal sources of meaning in older adults are associated with well-being and a greater sense of meaning [22]. Similarly, older adults who successfully achieve their goals, realize their values, and satisfy their needs at the highest level are happier and experience an increased sense of well-being [7]. Studies show that meaning is a valuable source of support for health and well-being. Individuals who shape their lives within the framework of meaning, integrity, and purpose they find in life have higher levels of well-being and positive emotions. Studies show that a meaningful life is associated with well-being [24]. Accordingly, our third hypothesis is that meaning and purpose would have a positive correlation with mental well-being (hypothesis 3).

### Freedom and Well-Being

According to Choice Theory, human beings are born with basic needs for freedom, entertainment, survival, power, love, and belonging [25]. According to this theory, the need for psychological freedom is for independence and autonomy [25]. This need is the ability to make choices, be creative, explore, and express themselves comfortably, as well as to have enough space to move freely and feel unrestricted while making choices and using free will. In this sense, freedom makes human life meaningful and purposeful. Individuals must feel a sense of freedom to achieve well-being [9]. Those who feel free report higher levels of well-being [26]. Similarly, the level of autonomy significantly contributes to well-being [27]. Values that promote well-being are called healthy values. Self-direction, which is used in a similar sense to freedom, is also positively related to well-being [28]. Therefore, we hypothesized that freedom would exhibit a positive correlation with mental well-being (hypothesis 4).

In summary, we aimed to investigate the predictive role of wisdom and life purpose in mental well-being among middle-aged to older adults using a multianalytical approach. More specifically, we tested 4 hypotheses: wisdom would exhibit a positive correlation with mental well-being (hypothesis 1), quality of life would exhibit a positive correlation with mental well-being (hypothesis 2), meaning and purpose would exhibit a positive correlation with mental well-being (hypothesis 3), and freedom would exhibit a positive correlation with mental well-being (hypothesis 4).

## Methods

### Participants and Procedure

To gather information from middle-aged to older adults living in Türkiye for the validation of the theoretical model and assessment of the proposed relationships, this cross-sectional study was conducted using an online survey package. Because of current happenings (ie, the COVID-19 pandemic) many adults consider face-to-face interactions risky; therefore, an online survey via Google Forms was the best method for collecting data. All participants were informed that taking part in the study was voluntary and that all information they provided would be kept private and anonymous and would only be used for research purposes. To prevent missing data, all questions and items were created in a required answer format.

The sample profile (n=377) shown in Table 1 indicates that 178 (52.3%) participants were women. Most respondents (n=244; 64.7%) were between 50 and 59 years old. The mean age was 59.09 (SD 9.08; range 50‐102) years. Of the participants, 146 (38.7%) had completed elementary school, 55 (14.6%) had completed middle school, 69 (18.3%) had completed high school, 23 (6.1%) had completed an associate degree, 77 (20.4%) had completed a bachelor’s degree, and 7 (1.9%) had a postgraduate degree. Most of the participants (n=293, 77.7%) had a lower to lower-middle income.

**Table 1.** Profile of the respondents (N=377).

Variables	Respondents, n (%)
Gender	
Woman	197 (52.3)
Man	180 (47.7)
Age group (y)	
50‐59	244 (64.7)
60‐69	80 (21.2)
70‐79	36 (9.5)
>80	17 (4.5)
Marital status	
Single	39 (10.3)
Married or living as a couple	335 (88.9)
Widowed, divorced, or separated	3 (0.8)
Spouse status	
Alive	317 (84.1)
Deceased	60 (15.9)
Number of children	
No child	35 (9.3)
1	27 (7.2)
2	96 (25.5)
3	89 (23.6)
4	63 (16.7)
5	29 (7.7)
>5	38 (10.1)
Income level	
Low income	150 (39.8)
Lower-middle Income	143 (37.9)
Upper-middle Income	62 (16.4)
High Income	22 (5.8)
Education level	
Primary education	146 (38.7)
Middle school	55 (14.6)
High school	69 (18.3)
College	23 (6.1)
Bachelor’s	77 (20.4)
Master’s	3 (0.8)
Doctorate	4 (1.1)

### Instruments

#### The Brief Self-Assessed Wisdom Scale

The scale was initially proposed by Fung et al [29] and adapted into Turkish for this study. The scale items were first translated into Turkish by 3 experts, who then reviewed and finalized the translated items by consensus. The adapted scale was then back-translated into English and compared with the original scale items. Reliability and validity assessments showed that the adapted scale displayed adequate psychometric properties. The scale consists of a single factor with 8 items, rated on a 6-point Likert-type scale (1 item was deleted after reliability analysis). An example scale item is “I have made important decisions throughout my life.” The Cronbach alpha value was 0.73 for the adapted scale and 0.81 for the original scale.

#### The Purpose in Life Scale

The scale was proposed by Crumbaugh and Maholick [30] to assess individuals’ sense of purpose and meaning in life and was adapted into Turkish by Kıraç [31]. The scale comprises 16 items distributed across 3 factors: quality of life, meaning and purpose, and freedom. Items in the scale were rated on a 7-point Likert-type scale. For instance, item 1, “I am usually,” was scored from 1 (“completely bored”) to 7 (“exuberant, enthusiastic”). Cronbach alpha value for the total scale was 0.91.

#### The Warwick-Edinburgh Mental Well-Being Scale

The scale was initially proposed by Tennant et al [32] and adapted into Turkish by Keldal [33]. It consists of a single factor with 14 items, scored on a 5-point Likert-type scale. For instance, item 1, “I have been feeling optimistic about the future,” was scored from 1 (“never”) to 5 (“always”). Cronbach alpha value for the scale was 0.89.

### Data Analysis

We used a confirmatory rather than an exploratory approach to validate and test the theoretical model. Thus, we decided that covariance-based structural equation modeling was preferable to partial least square structural equation modeling. However, the former cannot test nonlinear relationships between variables [34]. To address this limitation, we used a combined artificial neural network (ANN) and covariance-based structural equation modeling approach, enabling the identification of both linear and nonlinear relationships among the study variables [35]. This approach was used to test hypothesized linear relationships among latent variables and assess overall model fit. In addition, ANN analysis was conducted to capture potential nonlinear and complex relationships among the variables. This complementary approach allows covariance-based structural equation modeling to provide confirmatory evidence while ANN enhances predictive accuracy and explores relationships that may not be fully captured by linear modeling alone.

### Ethical Considerations

This study was approved by the institutional review board of Bandirma Onyedi Eylul University (IRB-01-2021/04). The study was conducted in accordance with the Declaration of Helsinki, and informed consent was obtained from all participants involved in the study.

## Results

### Reliability and Validity

We calculated composite reliability and Cronbach alpha to evaluate the internal consistency of the study constructs. The fourth item of the Brief Self-Assessed Wisdom Scale showed a Cronbach alpha of 0.669 if deleted, and its exclusion improved the reliability to 0.73. The alpha values ranged from 0.707 to 0.902, whereas composite reliability coefficients ranged from 0.711 to 0.906 (Table 2). Both coefficients exceeded the threshold value of 0.70 [36], indicating acceptable internal consistency. Moreover, factor analysis results showed factor loadings higher than 0.487 and communality values higher than 0.302. Some indicators exhibited factor loadings close to 0.50, indicating moderate reliability; these items were retained to preserve the constructs’ conceptual coverage.

**Table 2.** Reliability and validity results.

Constructs and indicators	Loading	Commonality	Cronbach α	Composite reliability
Mental well-being			0.902	0.906
Item 1	0.739	0.585		
Item 2	0.591	0.551		
Item 3	0.681	0.593		
Item 4	0.624	0.467		
Item 5	0.821	0.752		
Item 6	0.636	0.627		
Item 7	0.669	0.649		
Item 8	0.661	0.653		
Item 9	0.659	0.507		
Item 10	0.755	0.675		
Item 11	0.772	0.657		
Item 12	0.652	0.565		
Item 13	0.733	0.666		
Item 14	0.564	0.564		
Wisdom			0.730	0.730
Item 1	0.514	0.393		
Item 2	0.726	0.548		
Item 3	0.705	0.550		
Item 5	0.638	0.455		
Item 6	0.786	0.622		
Item 7	0.711	0.537		
Item 8	0.512	0.333		
Item 9	0.630	0.397		
Quality of life			0.834	0.836
Item 1	0.681	0.532		
Item 2	0.797	0.640		
Item 3	0.660	0.508		
Item 4	0.656	0.483		
Item 5	0.802	0.643		
Item 6	0.487	0.373		
Item 7	0.537	0.302		
Meaning and purpose			0.788	0.796
Item 8	0.680	0.581	.	
Item 9	0.571	0.534		
Item 10	0.702	0.582		
Item 11	0.554	0.408		
Item 12	0.726	0.561		
Item 13	0.573	0.632		
Item 14	0.604	0.504		
Freedom			0.707	0.711
Item 15	0.542	0.407		
Item 16	0.740	0.606		

### Measurement Model

To test the construct validity of the structural model, we performed confirmatory factor analysis, and the proposed relationships between the study variables were tested using covariance-based structural equation modeling. The measurement model demonstrated an acceptable fit to the data. The chi-square/degrees of freedom ratio was 2.23, and the root mean square error of approximation was 0.057 (90% CI 0.053‐0.061), both indicating reasonable model fit. Although goodness-of-fit index (0.823), adjusted goodness-of-fit index (0.790), comparative fit index (0.869), and Tucker-Lewis index (0.853) were slightly below conventional thresholds for excellent fit, parsimony-adjusted indices (parsimony normed fit index=0.700, parsimony comparative fit index=0.772) and information criteria (Akaike information criterion and Bayesian information criterion) support the adequacy of the model. Overall, results suggest that the measurement and structural models provide a satisfactory representation of the data (Table 3).

**Table 3.** Model fit statistics and indices.

Fit indices	Wisdom	Purpose in life	Mental well-being	Measurement model	Structural model	Threshold values
Chi-square (*df*)	41.2 (18)	237.7 (88)	159.4 (64)	1468.0 (658)	1621.0 (661)	—^a^
*P* value	.001	<.001	<.001	<.001	<.001	—
Chi-square/*df*	2.290	2.701	2.491	2.231	2.452	<3
Goodness-of-fit index	0.973	0.927	0.942	0.823	0.813	≥0.90
Adjusted goodness-of-fit index	0.946	0.887	0.905	0.790	0.780	≥0.80
Normed fit index	0.913	0.907	0.931	0.789	0.767	≥0.90
Tucker-Lewis index	0.919	0.916	0.939	0.853	0.827	≥0.90
Comparative fit index	0.948	0.938	0.957	0.869	0.845	≥0.90
Incremental fit index	0.949	0.939	0.957	0.871	0.847	≥0.90
RMSEA^b^ (LO90-HI90)^c^	0.059 (0.035-0.082)	0.067 (0.057-0.078)	0.063 (0.051-0.075)	0.057 (0.053-0.061)	0.062 (0.058-0.066)	≤0.08
Standardized root mean square residual	0.042	0.0540	0.0422	0.0628	0.1223	≤0.08

a Not applicable.

b RMSEA: root mean square error of approximation.

c LO90-HI90: 90% lower and upper intervals.

### Discriminant and Convergent Validity

As shown in Table 4, average variance extracted values ranged between 0.479 and 0.608. Although the average variance extracted for wisdom and quality of life was slightly below the recommended threshold of 0.50, their composite reliability values exceeded 0.70, indicating acceptable convergent validity [36]. Discriminant validity was assessed using the “heterotrait-monotrait ratio.”. As shown in Table 4, all heterotrait-monotrait ratio values were below the recommended threshold of 0.85, indicating satisfactory discriminant validity among the constructs [37].

**Table 4.** Discriminant validity (heterotrait-monotrait ratio) and average variance extracted (AVE).

Construct	AVE	Meaning and purpose	Mental well-being	Wisdom	Quality of life	Freedom
Meaning and purpose	0.543					
Mental well-being	0.608	0.47				
Wisdom	0.479	0.52	0.74			
Quality of life	0.497	0.39	0.68	0.71		
Freedom	0.507	0.44	0.62	0.66	0.63	

### Hypothesis Testing

Lateral collinearity and common method variance had been assessed before the hypotheses were tested. Harman single-factor test indicated that the first factor accounted for 32.36% of the total variance, suggesting that common method variance is unlikely to affect the results substantially. Furthermore, variance inflation factor values ranged from 1.347 to 2.616, indicating that multicollinearity among the predictors was low and did not pose a concern for the structural model estimate.

A 5000-sample bootstrapping technique (with a 95% CI) was used through SPSS AMOS (version 25; IBM Corp) to evaluate the structural model by calculating the standardized estimates (β), critical ratios or *t* values, and the coefficient of determination (*R*^2^). The results of hypothesis testing revealed a significant association between wisdom and mental well-being (β=0.248*; t*=4.732*; P*<.001). Further, mental well-being was significantly associated with quality of life (β=0.429; *t*=2.059; *P*=.04) and meaning and purpose (β=0.557; *t*=3.674; *P*<.001). These results supported first, second, and third hypotheses. However, no significant association was observed between mental well-being and freedom (β=−191, *t*=–1.383; *P*=.17). Thus, the fourth hypothesis was not supported. The results shown in Table 5 revealed that the theoretical model was meaningful, as the coefficient of determination (*R*^2^) for the 4 exogenous variables explained a total of 71% of the variance in the endogenous variable (*e*=0.12).

**Table 5.** Hypothesis testing results.

Hypothesis	Structural path	Standardized β	β (SE)		*t* value	*P* value	VIF^a^
1	Wisdom → mental well-being	0.248	0.225 (0.048)		4.732	.001	1.347
2	Quality of life → mental well-being	0.429	0.348 (0.169)		2.059	.04	2.616
3	Meaning and purpose → mental well-being	0.557	0.463 (0.126)		3.674	.001	2.101
4	Freedom → mental well-being	−0.191	−0.142 (0.103)		−1.383	.17	1.827

a VIF: variance inflation factor.

### The ANN Model

We further tested the proposed model using an ANN model to capture potential nonlinear relationships [38]. A multilayer perceptron model with one hidden layer and a single continuous output layer was used. The hidden layer used a hyperbolic tangent activation function, and the output layer applied an identity function to predict continuous mental well-being scores. All input covariates were standardized, and the dataset was divided into training (70%) and testing (30%) sets.

**Figure 2.**
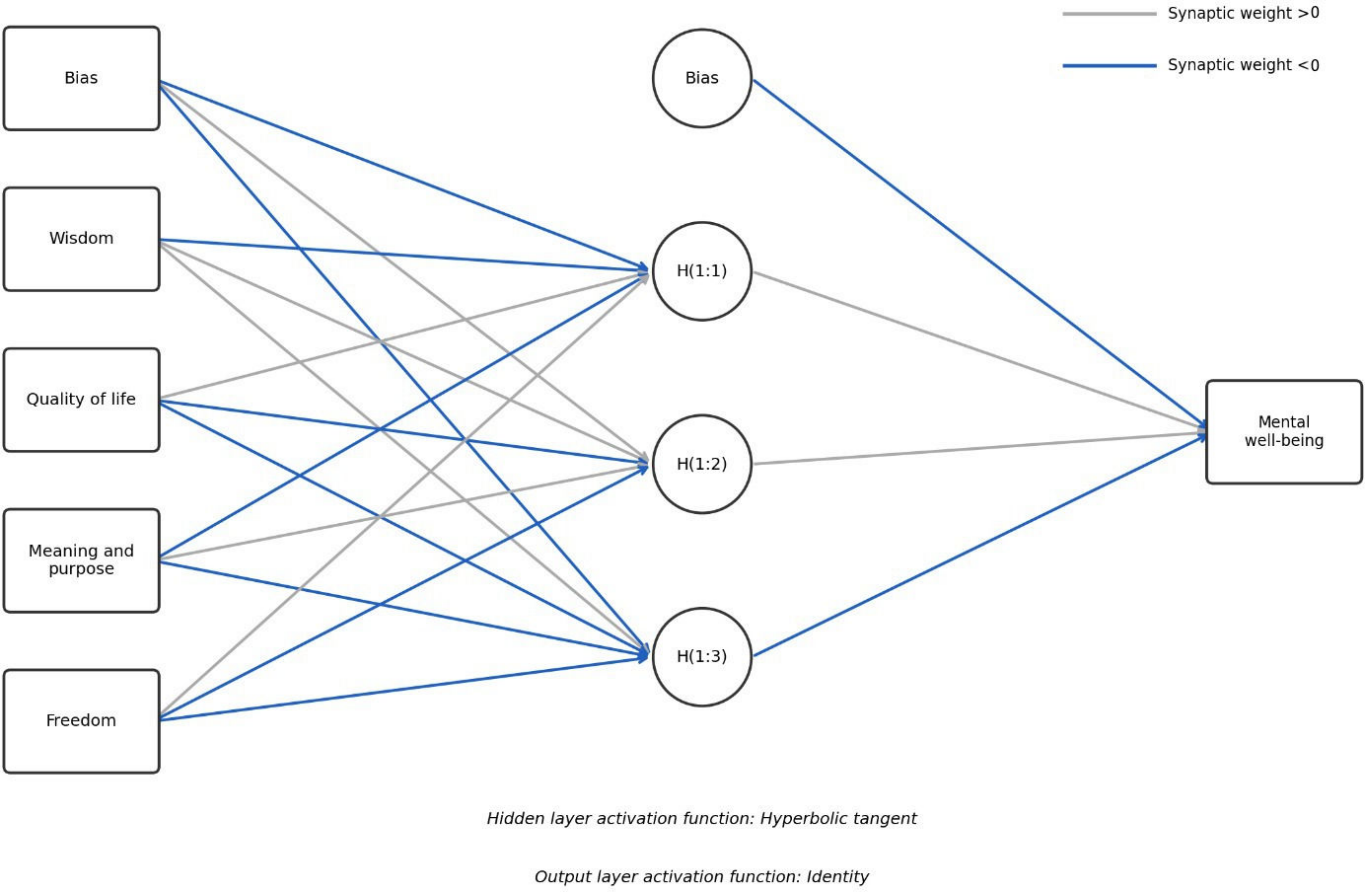
The artificial neural network (ANN) model. Hidden layer activation function: hyperbolic tangent. Output layer activation function: identity. H(1:1), H(1:2), and H(1:3) denote the first, second, and third neurons in the hidden layer, respectively.

As illustrated in Figure 2, the ANN model included an input layer representing the exogenous constructs, including wisdom, quality of life, meaning and purpose, and freedom, along with a bias term, followed by a hidden layer with 3 units and a continuous output layer for mental well-being. The predictive performance was evaluated using the sum of squared error and relative errors on the training and testing sets, which are appropriate metrics for continuous dependent variables. The model demonstrated good predictive performance, with sum of squares error and relative error values of 42.831 (training, relative error=0.335) and 20.191 (testing, relative error=0.499), indicating reasonable accuracy across the training and test sets.

Sensitivity analysis showed that quality of life was the most influential predictor (normalized importance=100%), followed by meaning and purpose (67.8%), wisdom (36.0%), and freedom (13.9%). These results highlight the relative contribution of each construct in predicting mental well-being. They are consistent with the covariance-based structural equation modeling results, in which wisdom, quality of life, meaning, and purpose showed significant positive effects. In contrast, freedom did not have a significant effect on mental well-being.

## Discussion

### Principal Findings

We aimed to investigate the predictive role of wisdom and life purpose in mental well-being among middle-aged to older adults using a multianalytical approach. More specifically, we tested 4 hypotheses: wisdom would exhibit a positive correlation with mental well-being (hypothesis 1), quality of life would exhibit a positive correlation with mental well-being (hypothesis 2), meaning and purpose would exhibit a positive correlation with mental well-being (hypothesis 3), and freedom would exhibit a positive correlation with mental well-being (hypothesis 4).

We found a significant association between wisdom and mental well-being (*P*<.05). Mental well-being was also significantly associated with quality of life, meaning, and purpose (*P*<.05). However, we did not find any significant association between mental well-being and freedom (*P*>.05). The results show that the theoretical model was meaningful. To detect nonlinear relationships among study variables, we used an ANN analysis. Sensitivity analysis indicated that the ranking of predictor importance is consistent with structural equation modeling results, confirming that quality of life, meaning and purpose, and wisdom are the main contributors to mental well-being, while freedom has minimal impact.

It is not possible to explain older adults’ well-being solely by living conditions such as the physical environment, socioeconomic status, physical health, and financial situation. Personality characteristics should also be considered to make a more realistic evaluation about their well-being [3]. For this purpose, we examined the relationships among mental well-being, perceptions of wisdom, quality of life, meaning and purpose in life, and freedom in individuals aged 50 years and older. Findings show a positive, significant relationship between mental well-being and wisdom, with wisdom being strongly associated with it. These findings were consistent with prior studies (eg, [15]).

Wisdom impacts well-being as a psychosocial developmental resource. Wisdom is the quality that an individual acquires from their communications, judgments, decision-making, and learning throughout their lives [19]. The level of wisdom increases depending on an individual’s life experiences. Given that older adults have more life experiences, their level of wisdom is expected to be deeper. According to the positive model’s assumption, there is a positive correlation between age and wisdom [13]. Although our findings confirm this assumption, old age may not always lead to wisdom. Whereas older age may be an advantage for wisdom, it is not the only sufficient condition, as the development of wisdom depends on various cognitive, emotional, cultural, and contextual factors [39]. Through a combination of these factors, wisdom among older adults can lead to mental well-being [14].

The emotion regulation and psychological balance that wisdom provides in older adults may not be satisfactory to sustain mental well-being. In addition, a high quality of life is necessary for mental well-being [40][46]. Physical functions, psychological states, social interactions, and occupational and economic situations affect individuals’ quality of life [41]. With age, older adults’ activities in society may be restricted by various psychosocial and economic factors, especially by chronic diseases [42][47]. In these circumstances, older adults’ quality of life declines.

Our results revealed a positive relationship between mental well-being and quality of life and that quality of life is significantly associated with mental well-being. A study conducted with older adults [43] yielded similar findings. In this context, mental well-being and quality of life are essential for individuals to establish more productive, stronger social relationships and make healthier decisions [33]. A decrease in the quality of life among older adults generates psychological problems, such as depression, anxiety, fear, and anger, as well as decreased well-being, life satisfaction, health status, and happiness [44]. Thus, it is important to provide quality-of-life standards with psychosocial and economic dimensions to maintain the mental well-being and positive aging of older adults.

One of the important psychological factors associated with mental well-being is meaning and purpose in life because it is related to a reduction in psychological burden, psychological distress, and mental illness (eg, anxiety, eating disorders, substance use, and depression) [22]. Psychological well-being and the meaning of life are considered the most important determinants of mental health and happiness [45]. Our results revealed a positive correlation between mental well-being and life purpose, with life purpose significantly associated with mental well-being. Studies of older adults have reported similar findings [46]. People who believe their lives are more meaningful experience better mental well-being and greater life satisfaction [22]. People aim to live their lives in a meaningful and purposeful way at every stage. While the purpose of life serves as a psychological buffer, particularly for people at the beginning of older ages, it is also an essential factor in sustaining their mental well-being.

Another factor that can make life meaningful and purposeful is freedom. Although the relationship between freedom and well-being is underexplored in the literature, an indirect study found a positive correlation [26]. However, our findings indicate no significant correlation between mental well-being and freedom. The nonsignificant impact of freedom may be explained by cultural and religious factors [47]. Türkiye is a society characterized by collectivist values and relatively high religiosity, where personal freedom is often understood in relation to family, social expectations, and religious norms. As a result, perceptions of freedom may differ from the individualistic conceptualization typically captured by the Purpose in Life Scale. This cultural context may have contributed to the lack of a significant association between the freedom dimension and mental well-being in this study.

Our findings have important implications for researchers and practitioners. Successful aging involves many psychological factors. This research contributes to the literature on which psychological factors should be considered to support successful aging. Furthermore, our results provide insight into preventive and reinforcing psychological factors to protect individuals’ psychological well-being during the preparatory stage for old age and to encourage them toward active aging.

### Limitations

Several psychological factors are essential for successful aging, including mental well-being, wisdom, quality of life, and purpose in life. This research shows that perceptions of wisdom, quality of life, meaning, and purpose in life are significantly associated with mental well-being among older people. Although the findings and implications of this study offer specific insights into the psychological factors associated with successful aging in later life, several limitations may limit the generalizability of the results.

Although Harman single-factor test indicated no severe common method bias, all variables were measured via a single self-report survey at one point, which may inflate correlations and should be considered when interpreting the results. Longitudinal or experimental studies are needed to mitigate these biases and clarify potential causal mechanisms underlying the proposed relationships. Another limitation concerns sample diversity. Most participants were aged 50 to 59 years and had relatively lower levels of education and income. In contrast, older adults (particularly those aged 70 years and above) and individuals from higher socioeconomic backgrounds were underrepresented. Therefore, future studies should recruit more diverse samples to enhance the generalizability of findings and provide a more comprehensive understanding of the relationships between wisdom, life purpose, and mental well-being across different segments of the population. Another limitation concerns the measurement of quality of life. In this study, this variable was derived from a subdimension of the Purpose in Life Scale rather than from a dedicated quality-of-life instrument. Because this subscale captures aspects of positive functioning and satisfaction, there may be conceptual overlaps with mental well-being, which may partly explain the relatively stronger associations observed. Future research could benefit from using established quality-of-life instruments to further strengthen construct differentiation. Additionally, the ANN model relied on a single 70:30 train-test split without repeated cross-validation, which may make the predictive performance estimates sensitive to sampling variability and limit their stability.

It should also be noted that broader cultural contexts may influence the perceptions of freedom. In societies where cultural norms, collective values, and beliefs play a significant role in shaping the meaning of life and individual choices, the interpretation of freedom may differ from that in more individualistic contexts. Future research could further explore how cultural frameworks influence perceptions of freedom and their potential associations with psychological well-being. Furthermore, the freedom dimension assessed by the Purpose of Life Scale may not fully capture the broader conceptualization of psychological freedom proposed by Choice Theory. Therefore, the interpretation of this dimension should be considered within the operational boundaries of the scale used in this study. Finally, the structural model did not include demographic or health-related covariates such as age, gender, education, income, marital status, or physical health, which may influence mental well-being. Future studies could include these variables to strengthen the robustness of the findings.

## Abbreviations

ANN: artificial neural network
